# Syndrome of the trephined: clinical spectrum, risk factors, and impact of cranioplasty on neurologic recovery in a prospective cohort

**DOI:** 10.1007/s10143-021-01655-6

**Published:** 2021-10-07

**Authors:** Lukas Sveikata, Lana Vasung, Amir El Rahal, Andrea Bartoli, Martin Bretzner, Karl Schaller, Armin Schnider, Béatrice Leemann

**Affiliations:** 1 Division of Neurorehabilitation, Department of Clinical Neurosciences, Geneva University Hospitals, Faculty of Medicine, University of Geneva, Geneva, Switzerland; 2 J. Philip Kistler Stroke Research Center, Department of Neurology, Massachusetts General Hospital, Harvard Medical School, Boston, MA USA; 3 Institute of Cardiology, Medical Academy, Lithuanian University of Health Sciences, Kaunas, Lithuania; 4 Boston Children’s Hospital, Harvard Medical School, Boston, MA USA; 5 Division of Neurosurgery, Department of Clinical Neurosciences, Geneva University Hospitals, Geneva, Switzerland; 6 Univ. Lille, Inserm, CHU Lille, U1172 - LilNCog (JPARC) - Lille Neurosciences & Cognition, 59000 Lille, France

**Keywords:** Cranioplasty, Decompressive craniectomy, Motor trephine syndrome, Neurologic recovery, Postoperative complications, Rehabilitation, Sinking skin flap, Stroke, Syndrome of the trephined

## Abstract

Syndrome of the trephined (SoT) is an underrecognized complication after decompressive craniectomy. We aimed to investigate SoT incidence, clinical spectrum, risk factors, and the impact of the cranioplasty on neurologic recovery. Patients undergoing a large craniectomy (> 80 cm^2^) and cranioplasty were prospectively evaluated using modified Rankin score (mRS), cognitive (attention/processing speed, executive function, language, visuospatial), motor (Motricity Index, Jamar dynamometer, postural score, gait assessment), and radiologic evaluation within four days before and after a cranioplasty. The primary outcome was SoT, diagnosed when a neurologic improvement was observed after the cranioplasty. The secondary outcome was a good neurologic outcome (mRS 0–3) 4 days and 90 days after the cranioplasty. Logistic regression models were used to evaluate the risk factors for SoT and the impact of cranioplasty timing on neurologic recovery. We enrolled 40 patients with a large craniectomy; 26 (65%) developed SoT and improved after the cranioplasty. Brain trauma, hemorrhagic lesions, and shifting of brain structures were associated with SoT. After cranioplasty, a shift towards a good outcome was observed within 4 days (*p* = 0.025) and persisted at 90 days (*p* = 0.005). Increasing delay to cranioplasty was associated with decreased odds of improvement when adjusting for age and baseline disability (odds ratio 0.96; 95% CI, 0.93–0.99, *p* = 0.012). In conclusion, SoT is frequent after craniectomy and interferes with neurologic recovery. High suspicion of SoT should be exercised in patients who fail to progress or have a previous trauma, hemorrhage, or shifting of brain structures. Performing the cranioplasty earlier was associated with improved and quantifiable neurologic recovery.

**Supplementary Information:**

The online version contains supplementary material available at 10.1007/s10143-021-01655-6.

**Figure:**
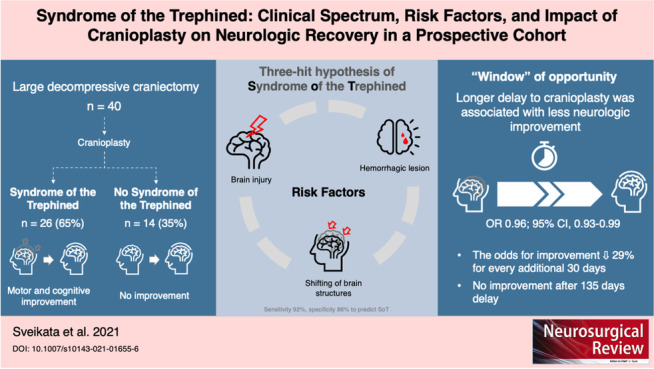
Graphical abstract

## Introduction

Syndrome of the trephined (SoT) is an underrecognized complication after decompressive craniectomy (DC) with poorly determined incidence ranging between 1 and 40% [16, 17, 29, 30, 34, 39]. SoT manifests by a delayed sensorimotor or cognitive worsening after craniectomy and is often associated with varying symptomatology, including headache, tinnitus, dizziness, fatigability, pain/discomfort at the site of craniectomy, feeling of apprehension, or depression [2, 15]. The wide range of clinical manifestations and absence of well-defined diagnostic criteria makes SoT a challenging diagnosis. One hallmark feature of SoT is a temporary improvement of symptoms in a supine position, termed orthostatic phenomena, which can help guide the diagnosis prior to cranioplast; whereas a definite improvement after a cranioplasty confirms SoT [5, 22, 35].

Furthermore, SoT is often associated with a sinking skin flap morphology, a radiologic [30] and clinical sign [47]. Although this association led to the development of new terminology for the syndrome (“sinking skin flap syndrome”), numerous findings in the literature indicate the existence of SoT in patients without sinking skin flap morphology[42]. Thus, there is growing evidence that the incidence of SoT might be underestimated because of a lack of detailed evaluation of subtle neurologic manifestations in the absence of a sinking skin flap. Since DC is increasingly utilized to treat refractory intracranial hypertension for various etiologies, including stroke [28] and traumatic brain injury (TBI) [20], there is an unmet demand for a better understanding of the post-craniectomy related complications.

Although known for almost a century [15, 47], the pathophysiology and risk factors predisposing to SoT are largely unknown [2]; as a result, SoT prediction remains challenging. Furthermore, many patients improve after cranioplasty without previous clinical worsening [19, 37], possibly due to a recovery impediment caused by insidious and underreported forms of SoT. Although emerging evidence suggests that earlier cranioplasty may improve the recovery of neurologic function [25], it remains unclear whether the improvement is related to insidious forms of SoT. Diagnostic methods allowing an early diagnosis of SoT and a better risk stratification are needed to inform clinical care and improve neurologic recovery.

To better understand the actual incidence and risk factors of SoT and assess the impact of cranioplasty timing on neurologic recovery, we performed a prospective observational study. We aimed to (1) evaluate the neurologic function immediately before and after the cranioplasty, (2) compare clinical and radiologic variables between patients with and without SoT, and (3) evaluate the association between the cranioplasty timing and improvement of disability immediately after the procedure.

## Materials and methods

### Design

From October 2012 to March 2017, we performed a prospective longitudinal cohort study of patients who underwent a large fronto-temporo-parietal DC, followed by cranioplasty at Geneva University Hospitals, Geneva, Switzerland. After clinical stabilization, patients transferred to the neurorehabilitation center were consecutively recruited. The participants underwent a comprehensive motor and neurocognitive assessment at admission, within 4 days before and after cranioplasty, and functional evaluation at 90 days.

The study was approved by the Geneva Regional Research Ethics Committee and was performed in accordance with the Declaration of Helsinki. All participants, or their next of kin, when applicable, provided informed consent. The study is in line with STROBE guidelines for observational research.

### Participants

Fifty-one patients referred to the neurorehabilitation center after DC were screened for eligibility. The inclusion criteria were (1) large DC (axial diameter > 12 cm) and (2) age 18 years or more. Exclusion criteria were (1) refusal to participate (*n* = 10) and (2) immediate severe complication after cranioplasty limiting neurologic assessment (*n* = 1). A total of 40 patients were included and completed the 90 days follow-up.

### Syndrome of the trephined diagnosis

SoT was defined as neurologic deterioration or failure to progress before cranioplasty and a rapid improvement of neurologic function within four days after the procedure. Alternative diagnoses were excluded before the cranioplasty, e.g., hydrocephalus, seizure, infection, or new-onset stroke. Based on neurologic findings and further clinical workup, two trained neurologists (L.S., B.L.) diagnosed SoT.

#### *“*A priori*” and “*a posteriori*” SoT*

Given cranioplasty’s therapeutic role, we stratified cases into “a priori” and “a posteriori” SoT. In “a priori”, a more severe form of SoT, patients developed new neurologic symptoms or deterioration before the cranioplasty and improved within 4 days after the cranioplasty; whereas in “a posteriori” SoT, patients failed to progress before the cranioplasty and improved within 4 days after the procedure.

### Data collection

Patient demographic, clinical, imaging, SoT-related symptoms, and cranioplasty-related complication data were collected.

#### Neurologic assessment, disability, and imaging biomarkers

All patients underwent a comprehensive neurologic, including cognitive, assessment at admission, within 4 days before and 4 days after the cranioplasty. The neurologic assessment included a standard neurologic examination and a specific battery of motor tests: Motricity Index [6], grip strength evaluation with Jamar hand dynamometer [4], Postural Assessment Scale for Stroke Patients [3], and qualitative gait independence score (0 – bedridden; 1 – walking with substantial assistance from a therapist and < 10 meter distance, 2 – walking with a therapist and > 10 meter distance; 3 – independent gait with auxiliary equipment; 4 – independent gait without auxiliary equipment). The cognitive evaluation covered multiple domains, including working memory (digit span forward), executive function and processing speed (Trail Making Test A and B [1], Regard’s 5-point non-verbal fluency test [27]), language (institutional 12 object naming test, and Token test [36]), and visuospatial function (Bells cancellation test [13] quantitatively categorized: 0 – no neglect, 1 – mild, 2 – moderate, 3 – severe neglect). The clinical improvement was defined as an improvement of the SoT-related symptoms or improvement in motor/cognitive evaluation performed within 4 days before and after the cranioplasty. A clinically significant change in neurologic function was defined using the reliable change indices (RCI). Using RCI in psychometric tests allows the determination of whether the change of scores in an individual is significant and is greater than occurring due to random measurement error alone [10].

The modified Rankin Scale (mRS) was used to measure the degree of disability within 4 days after the cranioplasty, with mRS 0–3 defined as a good outcome. We also recorded the mRS at 90 days to evaluate the long-term functional status.

**Fig. 1:**
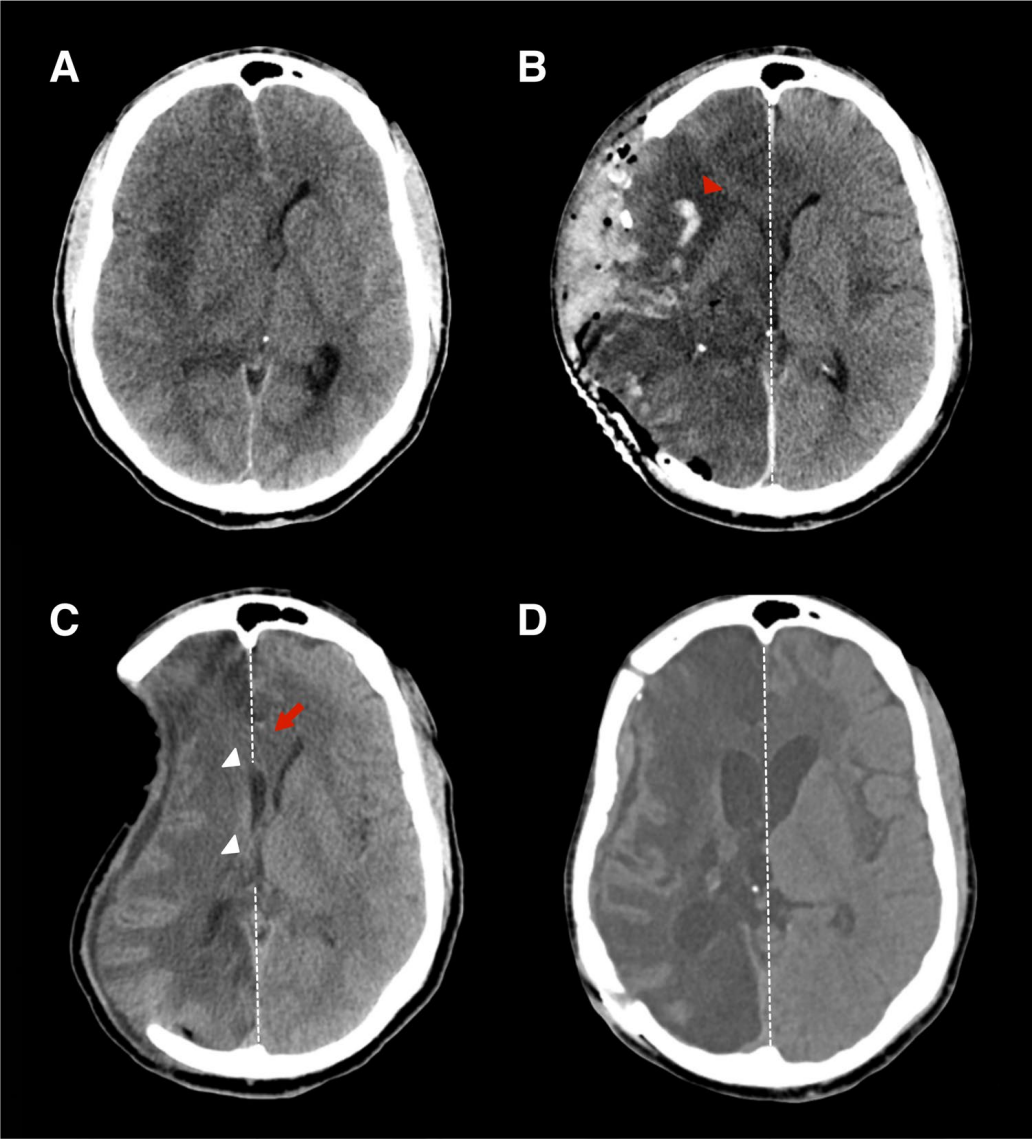
Serial imaging of SoT. *Legend*: **A** A right-handed individual without previous medical history underwent DC and right temporal lobectomy due to HSV-1 encephalitis induced brain edema. **B** The postoperative CT showed decompression of swollen brain tissue and a new intraparenchymal hemorrhage (red arrowhead). **C** Ten weeks after DC, the patient developed nausea and vomiting, worsening left-sided paresis, aphasia, decreased level of consciousness in a vertical position (GCS decrease from 11 to 8), and improvement to baseline in a supine position. Based on these clinical findings, an “a priori” SoT was diagnosed. CT imaging at 10 weeks showed the craniectomy site’s sinking appearance, paradoxical deviation of midline structures (red arrow), slit third ventricle, and AHLV (white arrowheads). **D** Cranioplasty was performed at week 11, resolving the radiologic signs of SoT and orthostatism. After cranioplasty, the motor function returned to baseline, consciousness improved (GCS 14), and the patient started to communicate in writing. Abbreviations: DC, decompressive craniectomy; HSV-1, herpes simplex virus type 1; GCS, Glasgow Coma Scale; AHLV, anterior horn of lateral ventricle; SoT, syndrome of the trephined

All patients underwent a head CT scan (Siemens Somatom Force or GE Discovery 750 HD) in a supine position. Imaging biomarkers and standard radiologic signs of the SoT were recorded as reported in our previous publication [42]. The following markers indicated a shift of brain structures: the sinking skin flap at the craniectomy site, deviation of the midline structures (Fig. 1), axial diameter, and slit-like third ventricle and anterior horn of the lateral ventricle. The paradoxical herniation of midline structures was marked with positive values, while deviation towards the craniectomy side was marked with negative values.

The craniectomy area was assumed to be an ellipse and calculated using the following equation:

$$A=(\pi ab)/4,$$

where *a* is the maximal diameter in the axial plane, and *b* is the coronal plane’s maximal diameter.

Lastly, we included the radiologic signs of ipsilateral hemorrhagic lesion defined as a composite variable of bleeding in the leptomeningeal compartment either pre- or post-craniectomy procedure: intraparenchymal hemorrhage, subarachnoid hemorrhage, blossoming contusion, or hemorrhagic transformation of ischemic stroke (Fig. 1). Subdural and epidural hemorrhages were not included as being outside pial and subarachnoid space they would not affect the perivascular drainage pathways [43]. All measurements were obtained using the Osirix software (Pixmeo Sarl, Geneva, Switzerland).

### Statistical analysis

Continuous variables were compared using Student’s *t*-test or Mann–Whitney *U* and categorical variables using Fisher’s exact or Pearson chi-square, as appropriate. First, we ran an exploratory univariate analysis to assess baseline imbalances between the SoT versus non-SoT groups and “a priori” versus “a posteriori” SoT. Exploratory pairwise comparisons were adjusted for false discovery rate (FDR) using the Benjamini–Hochberg procedure (FDR < 0.1).

Second, to examine the impact of multiple risk factors on SoT, we conducted a forward stepwise logistic regression with three independent variables to avoid overfitting. Variables with a significance of* p* < 0.1 in the group comparison analysis were used in univariate analysis; contributing variables with a significance of *p* < 0.05 remained in the multivariable model. The explained variance was measured using the Cox and Snell pseudo-*R*^*2*^ method and the predictive power with the area under the curve (AUC).

Third, the results of individual neurologic tests within 4 days before and 4 days after the cranioplasty were compared using the *t*-test and Wilcoxon signed ranks test, as appropriate. On an individual level, a significant improvement on neurologic tests was defined using RCI with 80% confidence intervals calculated as previously described [38] using the standard deviation of the test and its test–retest reliability index [36].

Fourth, Cochran’s *Q* test was used for ordinal shift analysis to compare the proportion of good neurologic outcome within 4 days before and after the cranioplasty.

Fifth, the association between disability improvement and delay to cranioplasty was evaluated using a logistic regression model, adjusting for age and baseline disability. *p* < 0.05 (2-tailed) was considered statistically significant unless stated otherwise. Data analyses were performed using SPSS version 26.0, GraphPad Prism v8.4.0, and R version 3.6.2.

## Results

### Cohort characteristics

Of 40 patients included in the analysis, 26 (65%) improved motor or cognitive function after the cranioplasty and were diagnosed with SoT. Fourteen patients (35%) developed clinically appreciable neurologic symptoms before the cranioplasty and improved within 4 days after the cranioplasty, termed “a priori” SoT, whereas twelve (30%) patients presented with failure to progress during the rehabilitation but improved within 4 days after the cranioplasty and were considered “a posteriori” SoT. The mean delay to cranioplasty was 112.8 ± 35.4 days. Table 1 presents the univariate comparison of clinical and imaging characteristics between SoT and non-SoT groups. There was a strong association between SoT and ipsilateral hemorrhagic lesions (*p* = 0.004) and shifting of brain structure (*p* < 0.001). There was also a weak association between SoT and TBI and an inverse association with ischemic stroke, although it did not maintain statistical significance after the FDR correction. Two patients, both in the SoT group, required placement of ventriculoperitoneal shunt for post-traumatic hydrocephalus prior to cranioplasty. The material used for cranioplasty did not differ between the groups and was autologous bone in 33 (82.5%), polyetheretherketone implant in 6 (15%), and titanium plating in 1 (2.5%).

**Table 1:** Clinical and imaging characteristics

Characteristics	All cohort (*n* = 40)	SoT (*n* = 26)	Non-SoT (*n* = 14)	*p* value*
Demographics and clinical				
Male, *n* (%)	26 (65)	17 (65)	9 (64)	1.000
Age, y (mean ± SD)	46.2 ± 12.6	46.2 ± 12.9	46.3 ± 12.5	0.983
Craniectomy area, cm2 (mean ± SD)	115.7 ± 18.5	114.0 ± 17.4	118.9 ± 20.6	0.428
Time to cranioplasty, days	112.8 ± 35.4	107.8 ± 41.0	122.1 ± 19.9	0.146
Left craniectomy, *n* (%)	19 (48)	15 (58)	4 (29)	0.105
Post-traumatic hydrocephalus, *n* (%)	2 (5)	2 (8)	0	0.533
Etiology for craniectomy, *n* (%)				
TBI	15 (38)	13 (50)	2 (14)	0.040
Ischemic stroke	13 (33)	5 (19)	8 (57)	0.031
Hemorrhagic stroke	8 (20)	6 (23)	2 (14)	0.689
SAH	1 (3)	0	1 (7)	0.350
Other	3 (8)	2 (8)	1 (7)	1.000
Imaging				
Ipsilateral hemorrhagic lesions, *n* (%)	31 (88)	**24 (92)**	**7 (50)**	0.004*
Shifting of brain structures, *n* (%)	24 (60)	**21 (81)**	**3 (21)**	< 0.001*
Sinking skin flap, *n* (%)	22 (55)	**19 (73)**	**3 (21)**	0.003*
Midline shift, mm (mean ± SD)	0.1 ± 3.8	**1.2 ± 3.9**	**-1.8 ± 2.8**	0.017*
Paradoxical herniation, *n* (%)	13 (33)	11 (42)	2 (14)	0.090
3rd ventricle axial diameter, mm (mean ± SD)	5.8 ± 3.4	5.3 ± 3.7	6.8 ± 2.5	0.073
Slit 3rd ventricle,* n* (%)	16 (40)	**14 (54)**	**2 (14)**	0.020*
AHLV diameter, mm (mean ± SD)	12.1 ± 6.8	10.9 ± 7.5	14.2 ± 4.6	0.103
Slit AHLV, n (%)	15 (38)	**14 (54)**	**1 (7)**	0.005*
Cranioplasty complications, *n* (%)				
Hemorrhagic complication	18 (45)	12 (46)	6 (43)	0.842
Requiring surgery	11 (28)	7 (27)	4 (29)	1.000
Surgical site infection	6 (15)	5 (19)	1 (7)	0.399

^*^ Significant values after false discovery rate correction are highlighted. Abbreviations: *SoT*, syndrome of the trephined; *SD*, standard deviation; *TBI*, traumatic brain injury; *SAH*, subarachnoid hemorrhage; *mRS*, modified Rankin scale; *AHLV*, anterior horn of lateral ventricle

### Risk factors

The unadjusted logistic regression model identified that TBI, ipsilateral hemorrhagic lesions, and shifting of brain structures were significantly associated with SoT (Table 2). In the multivariable regression model adjusted for age, we found that ipsilateral hemorrhagic lesions, and shifting of brain structures remained strong independent predictors of SoT, while TBI maintained a weak association with SoT. The model explained a significant proportion of the SoT occurrence variability (Cox and Snell *R*^2^ = 0.45) and had a strong predictive power (AUC = 0.93).

**Table 2:** Risk factors for SoT: logistic regression models

	Unadjusted			Adjusted^a^		
Variable	OR	95% CI	*p* value	OR	95% CI	*p* Value
Traumatic brain injury	6.0	3.1–76.8	0.03	9.9	0.9–112.2	0.065
Ipsilateral hemorrhagic lesion	12.0	2.0–71.4	0.006	22.1	1.4–354.0	0.029
Shifting of brain structures^b^	15.4	3.1–76.8	< 0.001	13.9	2.0–97.6	0.008

a Multivariable logistic regression model adjusted for age performed by stepwise method. Variance explained by *pseudo-R*^*2*^ = 0.45 (Cox and Snell), area under the curve = 0.93. Model *χ*^2^(1) = 24.14. ^b^ shifting of brain structures included at least one of the following: sinking skin flap, paradoxical herniation, slit-like third ventricle or anterior horn of the lateral ventricle. Abbreviations: *SoT*, syndrome of the trephined; *OR*, odds ratio; *CI*, confidence interval

### SoT clinical features and subtypes

All 26 SoT patients presented a rapid motor, cognitive improvement, or improvement in SoT symptoms (orthostatic phenomena, headache, vertigo, etc.) within 4 days after the cranioplasty. In Table 3, we present the most common clinical characteristics of SoT.

**Table 3:** Key clinical features of SoT

Characteristic	SoT cases (*n* = 26)
Days to key events after craniectomy	
Craniectomy to brain swelling resolution	45.9 ± 35.4
Craniectomy to SoT^a^	64.8 ± 24.8
Craniectomy to cranioplasty	107.8 ± 41.0
Clinical features	
Motor/sensitive	21 (81)
Motor impairment	21 (81)
Gait disturbance	6 (23)
Pyramidal signs	3 (12)
Sensitive deficit	1 (4)
Cortical functions	23 (88)
Executive function and attention/processing speed	14 (54)
Hemineglect	10 (39)
Language deficit	10 (39)
Altered mental state	4 (15)
Visual disturbance	3 (12)
Other symptoms	
Orthostatic phenomena	12 (46)
Headache	5 (19)
Nausea and vomiting	3 (12)
Seizures	3 (12)
Fatigue	2 (8)
Vertigo	1 (4)

^a^ Includes "a priori" SoT cases where neurologic deterioration was identified before the cranioplasty (*n* = 14). Values are mean ± SD or *n* (%) where appropriate. Abbreviations: *SoT*, syndrome of the trephined

When comparing the two modes of SoT presentation, the “a posteriori” SoT individuals were younger (38.8 ± 10.2 vs. 52.5 ± 11.8 years, *p* = 0.004) but did not differ in other clinical and demographic characteristics. The “a posteriori” SoT presented with less prominent radiologic features. Sinking skin flap (50% vs. 93%, *p* = 0.026) and paradoxical herniation (17% vs. 64%, *p* = 0.021) were less common in the “a posteriori” SoT group but did not differ in other radiologic features.

### Outcomes after the cranioplasty

#### Neurologic improvement

The baseline performance in motor and cognitive tests 4 days before the cranioplasty did not differ significantly between the SoT and non-SoT groups. However, there was a significant improvement in motor and cognitive performance in the SoT group within 4 days after the cranioplasty (Table 4). SoT patients improved significantly in Motricity Index, postural balance (PASS), gait, attention/processing speed and executive function (TMT A test and Regard’s 5-point verbal fluency test), and spatial neglect performance. Among “a priori” SoT patients, the cognitive and motor recovery was complete in 6/14 (43%) and partial in 8/14 (57%).

**Table 4:** Comparison of motor and cognitive performance in SoT patients 4 days before and after the cranioplasty

Evaluation in SoT group	n	Before cranioplasty	After cranioplasty	*p* value^a^
Motor function				
Motricity Index, mean ± SD	20	37.9 ± 30.0	51.1 ± 35.5	0.024*
Jamar, median (IQR)	11	4.0 (2.0–8.3)	5.0 (2.3–9.7)	0.362
PASS, median (IQR)	18	29.0 (23.3–32.8)	33.0 (29.5–35.0)	0.009**
Gait independence score, median (IQR)	25	3.0 (1.0–3.0)	3.0 (1.5–4.0)	0.024*
Pyramidal signs (%)	26	18 (69.2)	16 (61.5)	0.560
Cognitive function				
GCS, median (IQR)	25	15.0 (14.0–15.0)	15.0 (14.0–15.0)	1.0
TMT A, mean ± SD	9	140.9 ± 72.7	100.1 ± 47.7	0.015*
TMT B, mean ± SD	4	219.3 ± 90.9	185.3 ± 118.5	0.105
Digit span forward, median (IQR)	22	4.0 (0.8–6.0)	4.0 (0.8–6.3)	0.705
Regard’s 5-point non-verbal fluency test, mean ± SD	20	8.8 ± 7.8	10.8 ± 8.5	0.037*
12 object naming test, median (IQR)	25	11.5 (0–12.0)	11.0 (0–12.0)	0.180
Token test errors (language), median (IQR)	14	17.5 (0–20.0)	16.0 (0–20.0)	0.102
Spatial neglect severity score, median (IQR)	25	1.5 (0–3.0)	1.0 (0–2.0)	0.031*

^a^*t*-test or Wilcoxon signed ranks test, as appropriate. Abbreviations: *SD*, standard deviation; *Jamar*, grip strength evaluation with Jamar Hand Dynamometer; *IQR*, interquartile range; *PASS*, Postural Assessment Scale for Stroke; *GCS*, Glasgow Coma Scale; *TMT*, Trail Making Test. Significance level p < 0.05 (*) and p < 0.01 (**)

#### Change in disability

**Fig. 2:**
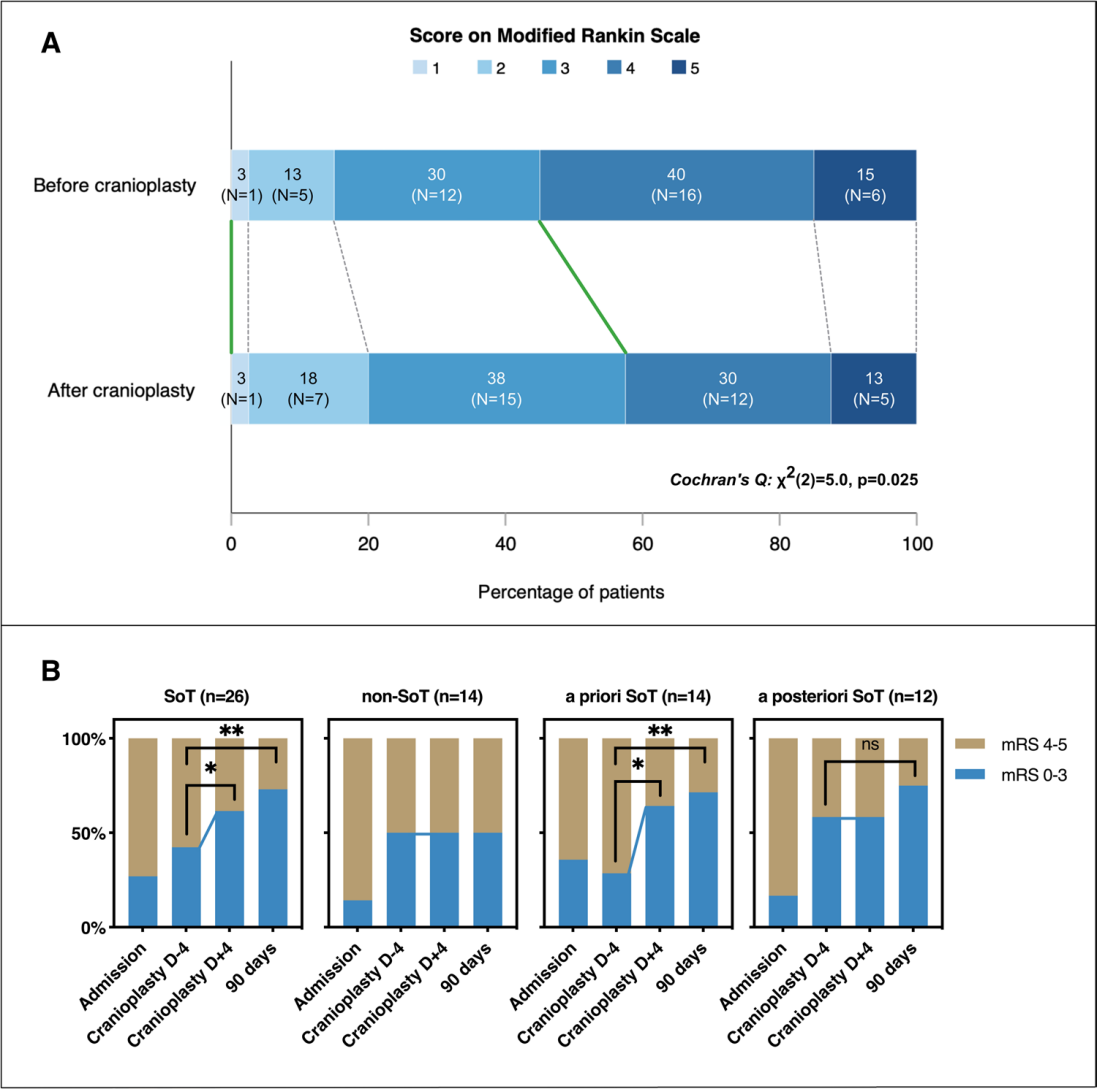
Impact of cranioplasty on neurologic outcome after cranioplasty. *Legend:* **A** Modified Rankin scale (mRS) score at 1–4 days before and 1–4 days after cranioplasty (*n* = 40). More patients presented a good neurological outcome (mRS 0–3) after the cranioplasty than before (*p* = 0.025; green lines). **B** The proportion of good neurological outcome (mRS 0–3) stratified by SoT severity. In the SoT group, there was a significant shift towards a good outcome within 4 days after the cranioplasty (62% vs. 42%, *p* = 0.025) that persisted at 90 days (73% vs. 42%, *p* = 0.005). Mean mRS improved in the SoT group within 4 days after the cranioplasty (3.4 ± 0.9 vs. 3.7 ± 0.8, *p* = 0.008). In the “a priori” SoT group, a similar shift towards good outcome (64% vs. 29%, *p* = 0.025) and improved mRS (3.3 ± 1.0 vs. 3.9 ± 0.9, *p* = 0.006) was observed. In the “a posteriori” SoT group, there was no significant change in the mRS within four days after the cranioplasty, but there was a trend towards a good outcome and a significant mean mRS improvement at 90 days (75% vs. 58%, *p* = 0.157, and 2.9 ± 1.1 vs. 3.4 ± 0.8, *p* = 0.007, respectively). Significance level *p* < 0.05 (*) and *p* < 0.01 (**). Abbreviations: SoT, syndrome of the trephined; D-4, 4 days before cranioplasty; D + 4, 4 days after cranioplasty, ns, non-significant

The neurologic improvement resulted in an improved disability (mRS) within 4 days after the cranioplasty in 7/26 (27%) SoT patients. Cochran’s *Q* test indicated a significant ordinal shift towards a good outcome after the cranioplasty (58% vs. 45%, *p* = 0.025; Fig. 2).

In the SoT group, there was a significant shift towards a good outcome within 4 days after the cranioplasty (*p* = 0.025) that was maintained at 90 days (*p* = 0.005, Fig. 2).

In the “a priori” SoT group, a similar shift towards good outcome (*p* = 0.025) and improved mRS (*p* = 0.006) was observed. In the “a posteriori” SoT group, the neurologic improvement did not result in  mean mRS change within 4 days after the cranioplasty. Nevertheless, there was a trend towards a good outcome (*p* = 0.157) and a significant mean mRS improvement at 90 days (*p* = 0.007) that was not observed in the non-SoT group.

### Delay to cranioplasty and neurologic recovery

**Fig. 3:**
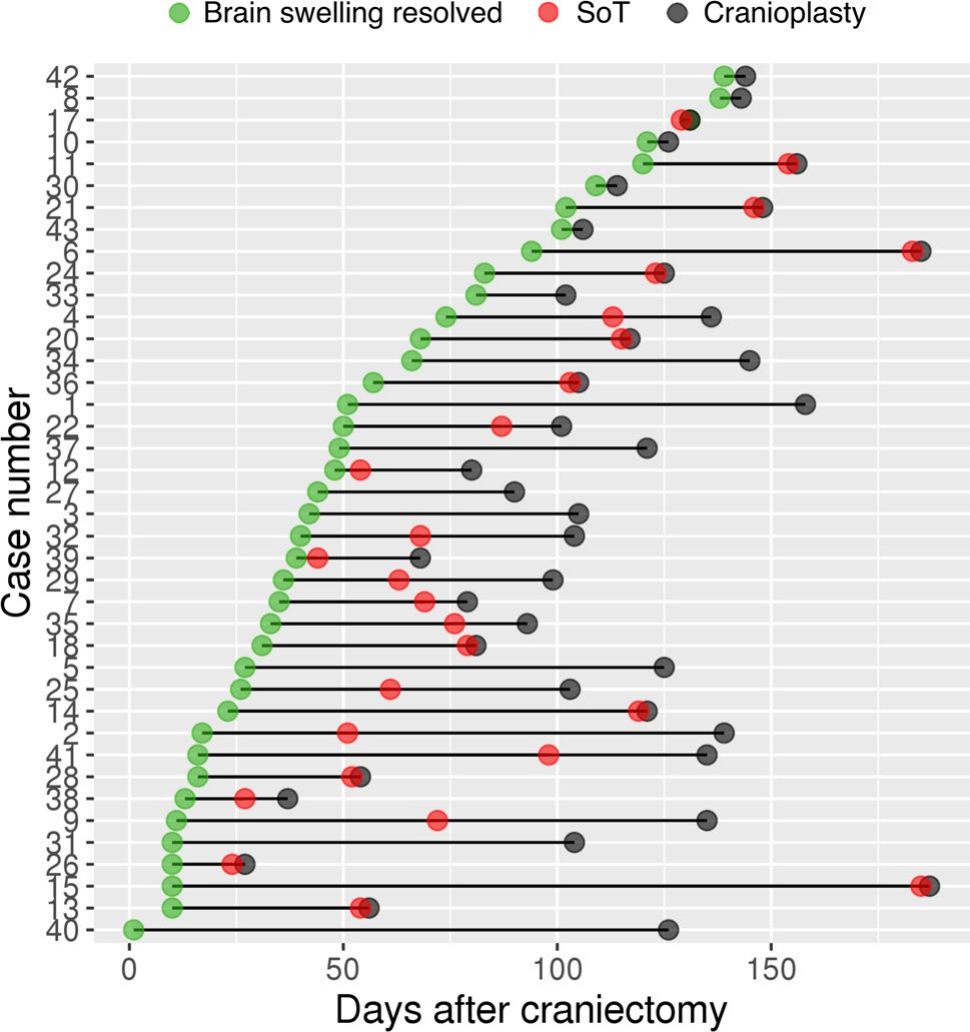
Delay to key events after the craniectomy. *Legend*: Days to brain swelling disappearance in the craniectomy site (green dot), SoT (red dot), and to cranioplasty (black dot) are indicated for individual patients. The non-overlapping red dots correspond to “a priori” SoT (clinical deterioration before cranioplasty), and overlapping red and black dots correspond to “a posteriori” SoT. “A posteriori” SoT was diagnosed when neurologic improvement was observed within four days after the cranioplasty

We observed a significant delay between the resolution of brain swelling at the craniectomy site (54.3 ± 40.4 days) and cranioplasty (112.8 ± 35.4; Fig. 3). The delay from edema disappearance to cranioplasty was longer than 1 month in 30 (75%) and longer than 2 months in 18 (45%) patients.

**Fig. 4:**
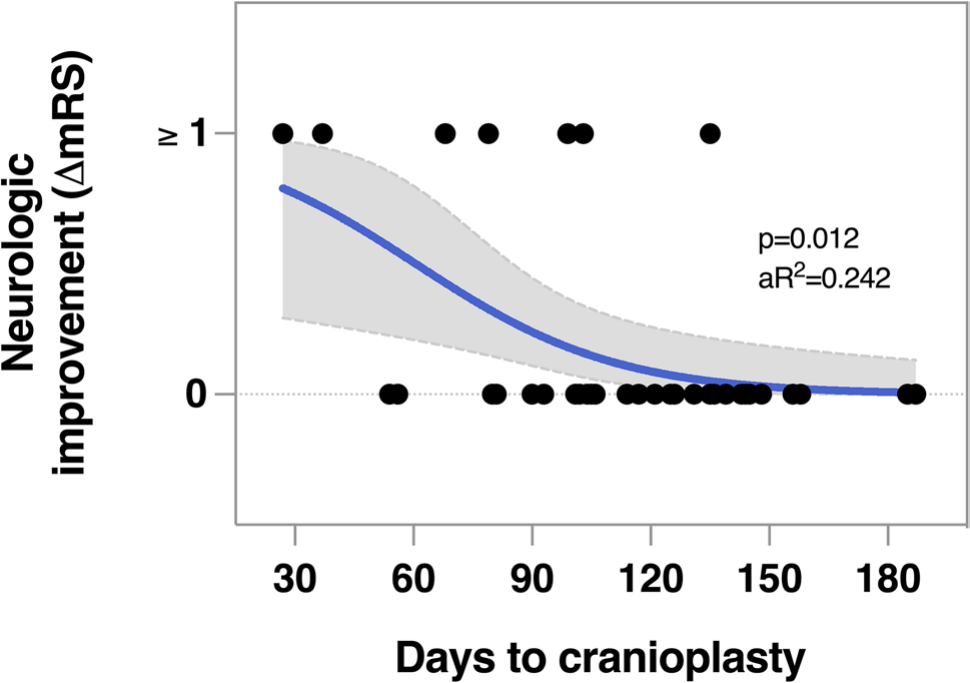
Shorter delay to cranioplasty was associated with better neurologic outcomes in a logistic regression model. *Legend:* Delay to cranioplasty (*β* =  − 0.04, *p* = 0.012) was negatively associated with neurologic improvement after cranioplasty adjusting for age and baseline disability. Variance explained by *R*^2^ = 0.24 (Cox and Snell), model *χ*^2^(1) = 8.62. Abbreviations: mRS, modified Rankin Scale, a*R*^2^, adjusted *R*^2^

Increasing delay to cranioplasty was independently associated with less neurologic improvement. The odds for improvement decreased by 4% for every additional day to cranioplasty after adjusting for age and baseline disability (OR = 0.96, 95% CI 0.93–0.99; *p* = 0.026; Fig. 4). An additional delay of 30 days decreased the odds to improve by 29%. No improvement was observed when the delay to cranioplasty was 135 days or longer.

## Discussion

The main finding of this study is the high incidence of SoT in patients with large craniectomies, suggesting that the condition is underreported [16, 17, 30, 34, 39]. Contrary to previous studies we have identified that 65% of patients suffered from SoT. A detailed prospective neurologic and radiologic assessment within 4 days before and after cranioplasty, allowed us to identify the complete spectrum of SoT. We then identified three independent risk factors contributing to SoT that may help stratify SoT risk in clinical practice and research. Third, we identified and quantified the impact of cranioplasty timing on neurologic recovery. Our findings suggest an association between earlier cranioplasty and improved neurologic recovery.

### Detailed neurologic assessment is instrumental in detecting SoT

A detailed neurologic and cognitive assessment within 4 days before and after the cranioplasty allowed us to detect milder forms of SoT that we termed the “a posteriori” SoT. “A posteriori” SoT manifested in slow rehabilitation progress followed by a measurable neurologic improvement within four days after the cranioplasty. The repeated neurologic testing after the cranioplasty improved diagnostic sensitivity and explains a significantly increased incidence of SoT. Our study adds to the growing body of literature demonstrating that a significant proportion of patients improve after cranioplasty even without evident neurologic worsening beforehand [19]. Our findings of an improved neurologic function in the “a posteriori” SoT group corroborate the observations that a proportion of patients improve after cranioplasty even without an apparent worsening beforehand. Thus, our findings suggest that “a priori” and “a posteriori” SoT constitute a clinical spectrum of SoT. Consequently, a significant proportion of patients after craniectomy are susceptible to develop SoT. Slow rehabilitation progress in craniectomized patients should alert clinicians to consider tailoring the cranioplasty timing to an individual patient.

The SoT diagnosis remains challenging due to the absence of robust diagnostic criteria. In the current study, the radiologic assessment revealed that 81% of the SoT group presented at least one sign of shifting brain structures, e.g., sinking skin flap, paradoxical midline shift, compressed lateral or 3^rd^ ventricle, but their diagnostic yield individually remained low. SoT manifested without the classical radiologic sign of sinking skin flap in 50% and without paradoxical herniation in more than 80% of the “a posteriori” SoT patients, corroborating the data from our previous study [42]. As a result, the absence of a sinking skin flap or paradoxical herniation does not exclude SoT, and careful clinical evaluation should guide the diagnosis. Conversely, radiologic signs of the brain structure shifting should warrant a careful repeated neurologic evaluation and to consider an expedited cranioplasty.

### Brain injury, hemorrhage, and shifting of the brain are risk factors for SoT

Our results suggest that hemorrhagic lesions in the leptomeningeal compartment and shifting of brain structures are associated with the development of SoT. Although TBI did not maintain a strong association in multivariable analysis, the association between TBI and SoT is intriguing, warranting confirmation in larger cohorts.

There was no significant confounding between the shift of brain structures and TBI or the hemorrhagic lesions and TBI. Consequently, we suggest a cumulative effect of these three risk factors on the development of SoT. Stiver et al. showed that brain contusions, together with abnormal cerebrospinal fluid (CSF) circulation, represent a risk factor for developing SoT [34]. Similarly, in a study evaluating a large registry including 43 SoT cases, Di Rienzo et al. found an association between TBI and SoT [29]. On the other hand, although our results suggest that ischemic stroke presents a lower risk for SoT, stroke etiology should not exclude SoT, as it has been previously shown to occur in stroke cohorts [30].

#### The three-hit hypothesis for developing SoT

Based on the study results, we suggest a three-hit hypothesis for the pathophysiology of SoT:

1. *Initial brain injury by TBI* causes a wide range of functional short- and long-term neurologic deficits associated with contusions [33] and diffuse axonal injury [32] with underlying healthy brain tissue that has the potential to recover.
2. *Cranial window results in brain structure shift and disturbs physiologic intracranial fluid dynamics.* The physical shift of brain structures and compression by a sinking skin flap causes blood flow disturbances [44], cerebral metabolism impairment [44, 48], and changes in the CSF flow [11, 42]. The loss of the brain’s rigid enclosing causes reduced pulse wave amplitude [24], which impairs intracranial fluid movement, including capillary blood flow, CSF circulation, and perivascular drainage [26].
3. *Hemorrhagic lesions further impair CSF production and clearance.* Increased atmospheric pressure and blood degradation products in the brain parenchyma [12] and subarachnoid space [14] impair CSF formation and clearance through blockage of arachnoid granulations by blood clots. Additionally, emerging evidence suggests that perivascular drainage plays an important role in the drainage of brain solutes [41]; thus, the impairment of perivascular drainage may additionally contribute to impaired brain fluids dynamics and SoT development [12, 23].

Interestingly, although recent studies have shown an association between craniectomy size and SoT [29, 39], we could not confirm these findings [29, 39]. In a study by Tarr et al., SoT incidence increased when craniectomy area reached 50 cm^2^ or more [39]. Although we did not find a difference in mean craniectomy area between the SoT and non-SoT groups, the inclusion of only large craniectomies in our study (mean craniectomy area 112.8 ± 35.4 cm^2^) limited the sensitivity to detect this association. However, our findings suggest that there might be a ceiling effect when SoT risk stops increasing after a certain threshold of craniectomy area is reached. As a result, large craniectomies may have been an additional contributor to the high incidence of SoT in our cohort.

### Effect of cranioplasty on neurologic symptoms and disability

All SoT patients improved in motor and cognitive function within 4 days after the cranioplasty confirming the SoT diagnosis [2]. In contrast, Honeybul et al. reported a 16% improvement rate in patients undergoing cranioplasty. However, their cohort did not include patients with worsening neurologic status before the cranioplasty (i.e., “a priori” SoT), arguably representing less severe SoT cases. According to the literature, the delay to improvement observed after cranioplasty varies from 1 to 4 days [2, 17, 39]. There is also evidence suggesting that cerebral perfusion abnormalities improve in a similar timeframe [35]. As a result, evaluating neurologic symptoms within 4 days in this study was considered optimal for increasing the SoT detection sensitivity.

Our results revealed that neurologic improvement after cranioplasty in SoT patients led to a significant improvement in the quality of life (i.e., decreased disability) and a shift towards good neurologic outcome. This study adds to the growing body of literature on the positive impact of cranioplasty on neurologic recovery.

### Does timing of the cranioplasty improve neurologic recovery?

We found an association between improved neurologic recovery and shorter delays to cranioplasty. Although our results are in line with the emerging evidence that cranioplasty may improve neurologic function, and earlier cranioplasty may enhance this effect [18, 25], the question of optimal timing for cranioplasty procedure remains a complex issue. Multiple factors are at play when determining the optimal timing. One of them, addressed in our study, is the resolution of brain swelling giving place to successfully restore the skull integrity.

Our study results suggest a potential window of opportunity to perform cranioplasty as early as two weeks after the craniectomy as soon as the brain swelling resides. Potential benefits from cranioplasty, such as improved postural blood flow [44], cerebrovascular reserve capacity, cerebral metabolism [44, 48], and CSF flow [9], offer compelling arguments in favor of an earlier cranioplasty. In line with these previous studies, intracranial pressure monitoring studies showed that cranioplasty restored the physiological intracranial pressure dynamics during changes from supine to vertical position [24]. Thus, earlier cranioplasty could improve the aptitude to perform rehabilitation activities in a physiologic vertical position. However, to fully understand the equation for optimal cranioplasty timing, multiple variables have to be considered, such as the cranioplasty-related complications [31], risk of infection [40], role of post-traumatic hydrocephalus [21], over-drainage related to ventriculoperitoneal shunting [21], pre-cranioplasty morbidity [7], bleeding diathesis, and conditions related to the initial etiology for a craniectomy.

The evidence for complication risk regarding the timing of cranioplasty is conflicting. In our clinical experience, cranioplasty is often performed three or more months after DC [45, 46]. Some authors suggest that delaying the cranioplasty beyond 2 or even 6 months after DC may reduce the risk of complications and surgical site infections [31, 40]. Contrary to these findings, a meta-analysis of 18 studies and 2254 patients did not show any difference in infection rates when cranioplasty was performed earlier or later than three months after DC [46]. Another meta-analysis of 1209 patients confirmed similar infectious and hemorrhagic complication rates regardless of timing [45]. However, they also found an increased risk of hydrocephalus in the early cranioplasty group (relative risk 2.67, 95% CI 1.24 – 5.73), highlighting the complex relation of CSF circulation and the timing of cranioplasty [45].

In addition, the time of onset of “a posteriori” SoT cannot be fully appreciated as this insidious form rarely presents the anticipated “red flags,” such as sinking skin flap, paradoxical herniation, or clinical features of orthostatic phenomena, headaches, or vertigo. Despite the common insidious onset of SoT, our findings suggest that some of the SoT symptoms might not be fully reversible, further adding to the argument in favor of an earlier cranioplasty. In line with our findings, in previous studies the proportion of complete recovery ranged from 34.6 to 78% [2, 29, 39]. It is possible that earlier cranioplasty could prevent SoT but it remains unclear if an earlier cranial repair may be beneficial in all craniectomized patients or a subgroup with higher SoT risk. Performing cranioplasty as soon as brain tissue edema resolves may be preferable as it could facilitate participation in rehabilitation [8] and improve long-term neurologic outcomes [18, 25]. Because of the varying delay to brain swelling resolution and growing observational evidence of neurologic improvement after cranioplasty [25], future studies should seek to risk-stratify patients and tailor the timing of the cranioplasty to an individual patient rather than perform it at a fixed delay.

The reasons for the delay between brain edema resolution and cranioplasty in our study are not entirely clear. However, some of the contributing factors might be administrative and logistical considerations in organizing the cranioplasty surgery. It is crucial to foresee a timely transfer to a neurosurgical center when it is not available in the rehabilitation center’s vicinity. Secondly, some delays may occur due to the logistical delays required to produce and deliver a personalized cranial implants, such as the custom-made polyetheretherketone flap. Thus, efforts should be made to establish an early collaboration between the rehabilitation and neurosurgical teams to streamline the logistical aspects of the cranioplasty procedure.

### Limitations

Despite prospective design, our study has several limitations. The relatively small sample size resulted in wide confidence intervals, signifying a low level of precision, and should be interpreted with caution. Due to the increased number of univariate analyses, we ran into the risk of type I error that we adjusted for using FDR corrections. Hence, our results should be regarded as hypothesis-generating, highlighting potential mechanisms and associations to be confirmed in future studies on SoT. Nevertheless, it is reasonable to provide an informed discussion based on these pathophysiological hypotheses and assumptions in an effort to build a model or apply advanced neuroimaging techniques such as perfusion-weighted imaging or glymphatic MRI, which could further explain clinical and research findings and help develop hypothesis-driven studies in the field. More imaging and histopathological studies are needed to unravel the mechanisms of SoT that could lead to improved neurologic recovery in this fragile patient population.

Similarly, due to the relatively small sample size and observational design, we could not control for multiple variables influencing the delay to cranioplasty. Our findings, although preliminary, suggest an association between earlier cranioplasty and improved neurologic recovery. Randomized trials with larger sample sizes are warranted [21] to explore this association, controlling for multiple confounding factors and effect modifiers. Furthermore, cranioplasty is known to carry a high risk of postoperative complications, and therefore, in some cases, the post-operative hemorrhagic complications might mask the effect of cranioplasty on neurologic recovery. Lastly, ten patients failed to consent, potentially contributing to consent bias, which is relatively low due to the random distribution of non-consenting patients.

## Conclusions

This prospective longitudinal study demonstrates that SoT manifests with a spectrum of cognitive and motor symptoms in more than half of the patients undergoing craniectomy, causing significant concern for neurorehabilitation. We present two distinct types of SoT, including failure to progress during the rehabilitation and neurologic deterioration prior to cranioplasty, both requiring clinicians to consider the SoT diagnosis. Our findings suggest that ipsilateral hemorrhagic lesions, shifting of brain structures, and brain lesions related to TBI are independently associated with the pathophysiology of SoT. Cranioplasty performed as soon as brain edema resolves could help prevent the development of SoT and improve neurologic recovery. However, further studies are needed to determine the optimal timing for cranial repair at an individual level.

## Supplementary Information

Below is the link to the electronic supplementary material.

Supplementary file1 (DOCX 50 KB)

## Data Availability

The data that support the findings of this study are available from the corresponding author, upon reasonable request.

## Abbreviations

SoT: Syndrome of the trephined
DC: Decompressive craniectomy
TBI: Traumatic brain injury
CSF: Cerebrospinal fluid
mRS: Modified Rankin scale
