# Supplementary material for: Syndrome of the trephined: clinical spectrum, risk factors, and impact of cranioplasty on neurologic recovery in a prospective cohort
Source: Neurosurg Rev. 2021 Oct 7;45(2):1431–43. doi: 10.1007/s10143-021-01655-6 (PMC8976790; doi:10.1007/s10143-021-01655-6)
Supplement: Supplementary file 1 — Supplementary file1 (DOCX 50 KB) [file 10143_2021_1655_MOESM1_ESM.docx]

STROBE Statement—checklist of items that should be included in reports of observational studies

|  | Item No. | Recommendation | Page  No. | Relevant text from manuscript |
| --- | --- | --- | --- | --- |
| Title and abstract | 1 | (*a*) Indicate the study’s design with a commonly used term in the title or the abstract | 2 | In a prospective longitudinal study, we examined 40 consecutive patients that underwent a large decompressive craniectomy and cranioplasty for diverse etiologies: traumatic brain injury (TBI), stroke, intracranial hemorrhage. |
|  |  | (*b*) Provide in the abstract an informative and balanced summary of what was done and what was found | 2 |  |
| Introduction | | | |  |
| Background/rationale | 2 | Explain the scientific background and rationale for the investigation being reported | 5 | The risk factors predisposing to SoT are unknown, and its prediction remains challenging.^6,7^ Furthermore, a proportion of patients improve after cranioplasty without previous clinical worsening. Performing cranioplasty as soon as brain tissue edema subsides is preferable as it could improve participation in rehabilitation^11^ and neurological outcome.^12,13^ Diagnostic methods allowing an early diagnosis and a better risk stratification could inform care management. |
| Objectives | 3 | State specific objectives, including any prespecified hypotheses | 5 | To better understand the incidence and risk factors of SoT and to assess the impact of cranioplasty timing on the neurological outcome, we performed a prospective longitudinal study. We aimed to (1) evaluate neurological function immediately before and after the cranioplasty, (2) compare clinical and radiologic variables between patients with and without SoT, and (3) evaluate the association between the cranioplasty timing and degree of disability after the procedure and at 90 days. |
| Methods | | | |  |
| Study design | 4 | Present key elements of study design early in the paper | 6 | From October 2012 to March 2017, we performed a prospective longitudinal cohort study of patients who underwent large fronto-temporo-parietal DC (axial diameter >12 cm), followed by cranioplasty at [BLINDED FOR REVIEW]. After clinical stabilization, patients transferred to the neurorehabilitation center were consecutively recruited. The participants underwent a comprehensive motor and neurocognitive assessment at admission, within four days before and after cranioplasty, and functional evaluation at 90 days. |
| Setting | 5 | Describe the setting, locations, and relevant dates, including periods of recruitment, exposure, follow-up, and data collection | 6 | See above. |
| Participants | 6 | (*a*) *Cohort study*—Give the eligibility criteria, and the sources and methods of selection of participants. Describe methods of follow-up. | 6 | All of the 51 patients referred to the neurorehabilitation center after DC were screened for eligibility. The inclusion criteria were: (1) large DC (axial diameter >12 cm) and (2) age 18 years or more. Exclusion criteria were: (1) refusal to participate (n=10) and (2) immediate severe complication after cranioplasty limiting neurological assessment (n=1). A total of 40 patients were included and completed 90 days follow-up. |
| Variables | 7 | Clearly define all outcomes, exposures, predictors, potential confounders, and effect modifiers. Give diagnostic criteria, if applicable | 7 | SoT was defined as neurological deterioration or failure to progress before cranioplasty not attributable to other complications, e.g.  hydrocephalus, seizure, or new strokes, and a rapid improvement within 1-4 days after cranioplasty. Based on neurological findings and further clinical workup, two trained neurologists (L.S., B.L.) diagnosed the SoT.  The modified Rankin Scale (mRS) was used to measure the degree of disability within four days and 90 days after cranioplasty with mRS 0-3 defining good outcome.^8,21^ |
| Data sources/ measurement | 8* | For each variable of interest, give sources of data and details of methods of assessment (measurement). Describe comparability of assessment methods if there is more than one group | 7 | All patients included in the study underwent comprehensive neurological, including cognitive, assessment at admission, within four days before and after cranioplasty. The neurological assessment included a standard neurological examination and a specific battery of motor and cognitive tests covering multiple domains, including working memory (digit span forward^15^), long-term memory (institutional 8-item verbal and non-verbal test^16^), executive function (Trail Making Test^17^), language (object naming test^18^, and Token test^19^), and visuospatial function (Bells cancellation test^20^).  All patients underwent a head CT scanner (Siemens Somatom Force or GE Discovery 750 HD) in a supine position. Imaging biomarkers and standard radiological signs of the SoT were recorded as reported in our previous publication.^22^ The following markers indicated a shift of brain structures: sinking skin flap at the craniectomy site, deviation of the midline structures (figure 2A-D), axial diameter, and slit-like appearance of the third ventricle and anterior horn of the lateral ventricle. |
| Bias | 9 | Describe any efforts to address potential sources of bias | 7, 8, 16 | All patients included in the study underwent comprehensive neurological, including cognitive, assessment at admission, within four days before and after cranioplasty.  Subdural and epidural hemorrhages were not included as being outside pial and subarachnoid space they would not affect the perivascular drainage pathways.^23^  Second, to examine the impact of multiple risk factors on SoT, we conducted a forward stepwise logistic regression with up to three independent variables to avoid overfitting. SoT diagnostic score ranging from 0 to 3 was developed, dividing the beta coefficients by a common denominator. To cross-validate the score, we used a new logistic regression model derived from randomly selected 35 patients and cross-tested the score in two steps: five remaining and five additional randomly selected cases. Third, Cochran's Q test was used for ordinal shift analysis to compare good neurological outcomes before and after cranioplasty. Fourth, groups were stratified by SoT severity ('a priori' and 'a posteriori' SoT). Fifth, the association between disability improvement and delay to cranioplasty was evaluated using a logistic regression model, adjusting for age and baseline disability.  Third, ten patients failed to consent, potentially contributing to consent bias, which is relatively low due to the random distribution of non-consenting patients. |
| Study size | 10 | Explain how the study size was arrived at | 6 | All of the 51 patients referred to the neurorehabilitation center after DC were screened for eligibility. The inclusion criteria were: (1) large DC (axial diameter >12 cm) and (2) age 18 years or more. Exclusion criteria were: (1) refusal to participate (n=10) and (2) immediate severe complication after cranioplasty limiting neurological assessment (n=1). A total of 40 patients were included and completed 90 days follow-up. |

Continued on next page

| Quantitative variables | 11 | Explain how quantitative variables were handled in the analyses. If applicable, describe which groupings were chosen and why | 6 | Continuous variables were compared using Student t-test or Mann-Whitney U and categorical variables using Fisher's exact or Pearson Chi-square, as appropriate. First, we assessed baseline imbalances between the SoT and non-SoT groups. *P*<0.05 (2-tailed) was considered statistically significant. |
| --- | --- | --- | --- | --- |
| Statistical methods | 12 | (*a*) Describe all statistical methods, including those used to control for confounding | 7, 9, 14 | Patient demographic, clinical, and imaging data were collected.  Second, to examine the impact of multiple risk factors on SoT, we conducted a forward stepwise logistic regression with up to three independent variables to avoid overfitting.  Our results suggest that TBI, hemorrhagic lesions in the leptomeningeal compartment, and shifting of brain structures are risk factors for the development of SoT. There was no significant confounding between the shift of brain structures and TBI or the hemorrhagic lesions and TBI. Our results suggest that there is a cumulative effect of these risk factors on the development of SoT; thus, we developed a diagnostic SoT risk score with a high predictive value. |
|  |  | (*b*) Describe any methods used to examine subgroups and interactions | 9 | First, we assessed baseline imbalances between the SoT and non-SoT groups and identified variables that were statistically significant between the two groups (p<0.05). Odds ratio and 95% confidence intervals are reported. |
|  |  | (*c*) Explain how missing data were addressed | n/a |  |
|  |  | (*d*) *Cohort study*—If applicable, explain how loss to follow-up was addressed  *Case-control study*—If applicable, explain how matching of cases and controls was addressed  *Cross-sectional study*—If applicable, describe analytical methods taking account of sampling strategy | 6 | All of the 51 patients referred to the neurorehabilitation center after DC were screened for eligibility. The inclusion criteria were: (1) large DC (axial diameter >12 cm) and (2) age 18 years or more. Exclusion criteria were: (1) refusal to participate (n=10) and (2) immediate severe complication after cranioplasty limiting neurological assessment (n=1). A total of 40 patients were included and completed 90 days follow-up. |
|  |  | (*e*) Describe any sensitivity analyses | 9 | Fourth, groups were stratified by SoT severity ('a priori' and 'a posteriori' SoT). Fifth, the association between disability improvement and delay to cranioplasty was evaluated using a logistic regression model, adjusting for age and baseline disability. |
| Results | | | | |
| Participants | 13* | (a) Report numbers of individuals at each stage of study—eg numbers potentially eligible, examined for eligibility, confirmed eligible, included in the study, completing follow-up, and analysed | 6 | All of the 51 patients referred to the neurorehabilitation center after DC were screened for eligibility. The inclusion criteria were: (1) large DC (axial diameter >12 cm) and (2) age 18 years or more. Exclusion criteria were: (1) refusal to participate (n=10) and (2) immediate severe complication after cranioplasty limiting neurological assessment (n=1). A total of 40 patients were included and completed 90 days follow-up. |
|  |  | (b) Give reasons for non-participation at each stage | 6 | See above. |
|  |  | (c) Consider use of a flow diagram | n/a | Flow diagram was considered redundant for this study as inclusion criteria were stated in the main text. |
| Descriptive data | 14* | (a) Give characteristics of study participants (eg demographic, clinical, social) and information on exposures and potential confounders | 10 | Table 1 presents the comparison of clinical and imaging characteristics between SoT and non-SoT groups. The SoT group had significantly more TBI (50% vs. 14%, p=.040), less ischemic stroke (19% vs. 57%, p=.031), and more often had markers of brain structure shift (81% vs. 21%, p<0.001). The SoT group had a significantly longer hospitalization in rehabilitation facilities than the non-SoT (228.7 ± 136.6 vs. 143.2 ± 69.7 days, p=.044). |
|  |  | (b) Indicate number of participants with missing data for each variable of interest | 10 | No missing values, the group sizes are presented in Table 1. |
|  |  | (c) *Cohort study*—Summarise follow-up time (eg, average and total amount) | 10 & Table 1 | 90 days follow-up presented in Table 1. Total follow-up: The SoT group had a significantly longer stay in rehabilitation facilities than the non-SoT (228.7 ± 136.6 vs. 143.2 ± 69.7 days, p=0.044). |
| Outcome data | 15* | *Cohort study*—Report numbers of outcome events or summary measures over time | 10 | Out of 40 patients included in the analysis, 26 (65%) were diagnosed with SoT. Fourteen patients (35%) developed 'a priori' and 12 (30%) 'a posteriori' SoT. |
| Main results | 16 | (*a*) Give unadjusted estimates and, if applicable, confounder-adjusted estimates and their precision (eg, 95% confidence interval). Make clear which confounders were adjusted for and why they were included | 10 | Our exploratory analysis revealed that the odds ratio (OR) of SoT was six times higher after TBI. Similar to craniectomy’s etiology, shifting brain structures and ipsilateral hemorrhagic lesions were also significantly more frequent in the SoT group (Table 1). The regression model explained a significant proportion of the variability of the SoT occurrence (Nagelkerge R^2^=0.62; table 2). |
|  |  | (*b*) Report category boundaries when continuous variables were categorized | 8 | The paradoxical herniation of midline structures was marked with positive values, while deviation towards the craniectomy side was marked in negative values. |
|  |  | (*c*) If relevant, consider translating estimates of relative risk into absolute risk for a meaningful time period | n/a |  |

Continued on next page

| Other analyses | 17 | Report other analyses done—eg analyses of subgroups and interactions, and sensitivity analyses | 11 | Seven patients (27%) from the SoT group improved the mRS score within four days post-cranioplasty, and none presented a worsening. Cochran’s Q test indicated a significant shift towards good outcome after cranioplasty (58% vs 45%; χ^2^(2)=5.0, p=.025; figure 3A).  In the SoT group, there was a significant shift towards good outcome within four days after cranioplasty and at 90 days (62% vs. 42%, p=.025, and 73% vs. 42%, p=.005, respectively) (figure 3B). There was a significant improvement in mean mRS in the SoT group (3.4 ± 0.9 vs. 3.7 ± 0.8, standard error of the mean [SEM]=0.1, p=.008).  In the 'a priori' SoT group, a similar shift towards good outcome (64% vs. 29%, p=.025) and improved mRS (3.3 ± 1.0 vs. 3.9 ± 0.9 [SEM=0.2], p=.006) was observed.  In the 'a posteriori' SoT group, the mean mRS did not change 1-4 days after the cranioplasty but there was a trend towards good outcome and a significant mean mRS improvement at 90 days (75% vs. 58%, p=.157, and 2.9 ± 1.1 vs. 3.4 ± 0.8 [SEM=0.2], p=0.007, respectively). |
| --- | --- | --- | --- | --- |
| Discussion | | | | |
| Key results | 18 | Summarise key results with reference to study objectives | 13 | Contrary to previous studies we found a significantly higher incidence of SoT in patients with large craniectomies.^1–4^ Using a detailed neurologic and radiologic assessment before and within four days after cranioplasty, we successfully diagnosed SoT in 65% of study participants. We then identified three risk factors and developed a SoT diagnostic score that has the potential to guide clinical management. Third, we identified and quantified the impact of cranioplasty timing on neurological recovery. Our findings suggest that SoT score could warrant an earlier cranioplasty and serve as a basis for individualized recommendations for cranioplasty timing. |
| Limitations | 19 | Discuss limitations of the study, taking into account sources of potential bias or imprecision. Discuss both direction and magnitude of any potential bias | 16-17 | Our study has several potential limitations. First, the large confidence intervals suggest a low level of precision and should be interpreted with caution. Future studies with larger sample sizes are needed to validate our findings. Second, cranioplasty-related complications might have masked the effect of cranioplasty on neurological improvement. Third, ten patients failed to consent, potentially contributing to consent bias, which is relatively low due to the random distribution of non-consenting patients. |
| Interpretation | 20 | Give a cautious overall interpretation of results considering objectives, limitations, multiplicity of analyses, results from similar studies, and other relevant evidence | 18 | This prospective longitudinal study demonstrates that SoT manifests in more than half of the patients undergoing craniectomy causing significant concern for neurorehabilitation. Our findings suggest that TBI, ipsilateral hemorrhagic lesions, and shifting of brain structures play a pivotal role in the development of SoT. Based on these risk factors, we suggest a simple to use SoT score that can warrant an earlier cranioplasty. Cranioplasty performed as soon as brain edema resolves could improve neurological recovery. |
| Generalisability | 21 | Discuss the generalisability (external validity) of the study results | 9, 16 | To cross-validate the score, we used a new logistic regression model derived from randomly selected 35 patients and cross-tested the score in two steps: five remaining and five additional randomly selected cases.  Our study has several potential limitations. First, the large confidence intervals suggest a low level of precision and should be interpreted with caution. Future studies with larger sample sizes are needed to validate our findings |
| Other information | |  | | |
| Funding | 22 | Give the source of funding and the role of the funders for the present study and, if applicable, for the original study on which the present article is based | 19 | This study was in part supported by a research grant from the Swiss National Science Foundation (P2GEP3_191584 (L.S.)) and conducted with support from Harvard Catalyst \| The Harvard Clinical and Translational Science Center (National Center for Advancing Translational Sciences, National Institutes of Health Award UL 1TR002541) and financial contributions from Harvard University and its affiliated academic healthcare centers. |

*Give information separately for cases and controls in case-control studies and, if applicable, for exposed and unexposed groups in cohort and cross-sectional studies.

**Note:** An Explanation and Elaboration article discusses each checklist item and gives methodological background and published examples of transparent reporting. The STROBE checklist is best used in conjunction with this article (freely available on the Web sites of PLoS Medicine at http://www.plosmedicine.org/, Annals of Internal Medicine at http://www.annals.org/, and Epidemiology at http://www.epidem.com/). Information on the STROBE Initiative is available at www.strobe-statement.org.
